# Topical Application of Ozonated Oils for the Treatment of MRSA Skin Infection in an Animal Model of Infected Ulcer

**DOI:** 10.3390/biology10050372

**Published:** 2021-04-26

**Authors:** Vanessa Silva, Cecília Peirone, Rosa Capita, Carlos Alonso-Calleja, José A. Marques-Magallanes, Isabel Pires, Luís Maltez, José Eduardo Pereira, Gilberto Igrejas, Patrícia Poeta

**Affiliations:** 1 Microbiology and Antibiotic Resistance Team (MicroART), Department of Veterinary Sciences, University of Trás-os-Montes and Alto Douro (UTAD), 5000-801 Vila Real, Portugal; vanessasilva@utad.pt; 2 Department of Genetics and Biotechnology, University of Trás-os-Montes and Alto Douro, 5000-801 Vila Real, Portugal; gigrejas@utad.pt; 3 Functional Genomics and Proteomics Unit, University of Trás-os-Montes and Alto Douro (UTAD), 5000-801 Vila Real, Portugal; 4 Associated Laboratory for Green Chemistry (LAQV-REQUIMTE), University NOVA of Lisboa, 2829-516 Caparica, Portugal; 5 Veterinary and Animal Research Centre, Associate Laboratory for Animal and Veterinary Science (AL4AnimalS), University of Trás-os-Montes and Alto Douro (UTAD), 5000-801 Vila Real, Portugal; ipires@utad.pt (I.P.); lmaltez@utad.pt (L.M.); jeduardo@utad.pt (J.E.P.); 6 Department of Veterinary Sciences, University of Trás-os-Montes and Alto Douro (UTAD), 5000-801 Vila Real, Portugal; cpeirone@chtmad.min-saude.pt; 7 Centro Hospitalar de Trás-os-Montes e Alto Douro E.P.E., 5000-185 Vila Real, Portugal; 8 Centre for the Research and Technology of Agro-Environmental and Biological Sciences (CITAB), UTAD, 5000-801 Vila Real, Portugal; 9 Department of Food Hygiene and Technology, Veterinary Faculty, University of León, 24071 León, Spain; rosa.capita@unileon.es (R.C.); carlos.alonso.calleja@unileon.es (C.A.-C.); 10 Institute of Food Science and Technology, University of León, 24071 León, Spain; 11 Critical Care Department, University Hospital Braga, 4710-243 Braga, Portugal; jamarques@povisa.es

**Keywords:** MRSA, antimicrobial resistance, ozone, ozonated oil, in vivo, diabetic foot ulcer

## Abstract

**Simple Summary:**

Methicillin-resistant *Staphylococcus aureus* is often found in skin lesions infected in particular in diabetic foot ulcers, in which the prevalence can reach 40%. Clinical methicillin-resistant *Staphylococcus aureus* is generally resistant to most classes of antibiotics and therefore it is necessary to develop new antimicrobial agents. Ozone has a recognized bactericidal activity and has been widely used as a clinical therapeutic agent for chronic wounds, such as ulcers and other injuries, due to its ability to heal wounds. This is a preliminary study that reports the effectiveness of ozonated oils on the eradication of skin infection in vivo. The study provides further insights into the antibacterial effect of ozone in infected skin ulcers in diabetic rats and also, the potential wound healing effect of ozonated oils. Furthermore, this is the first study investigating the efficacy of ozone in the eradication of diabetic foot ulcers infected by methicillin-resistant *Staphylococcus aureus*. Our results indicate that topical application of ozonated oils infected skin in rats have significant antimicrobial activity as well as wound healing capacity. Achieving the healing of infected diabetic foot wounds has become a challenge and ozonated oils may be used to treat these infections.

**Abstract:**

Diabetic foot ulcers are a common cause of morbidity in diabetic patients. One of the main pathogens found in these ulcers is methicillin-resistant *Staphylococcus aureus* (MRSA). MRSA often carries resistance to several classes of antibiotics and their infections are becoming harder to treat. Therefore, new alternatives are urgently needed. Thus, this study aimed to investigate the capacity of topical ozonated oil application on the treatment of early-stage skin infected with MRSA in an animal model. Ozonated oil was prepared from a mixture of oils subjected to a gas stream of O_2_/O_3_ mixture. Sixteen Wistar rats were inoculated by an intradermic injection of MRSA suspension, producing an abscess lesion. After 3 days, the skin epidermis was removed to open the wound. Group 1 received an application of oil mixture without ozone treatment and Group 2 received an application of ozonated oil. After the treatment period, skin was collected, colony-forming units (CFU) of bacteria were quantified and the histological analysis of the skin was carried out. Skin samples from the control 1 and 2 had a bacterial load was of 1.1 × 10^5^ and 5.7 × 10^3^ CFU/mL, respectively. Group 2 showed better wound healing from mild to moderate epidermal regeneration. Topical application of ozonated vegetable oil in MRSA-infected skin in rats showed a small reduction of the bacterial load and better wound healing.

## 1. Introduction

Diabetes belongs to the group of metabolic diseases, characterized by high blood glucose levels, and is one of four priority non-communicable diseases [1]. Globally, the prevalence of diabetes is increasing, and it was estimated that in 2014, 422 million adults were living with this disease which is nearly double the number of people with diabetes in 1980 [1]. Still, it is expected that the prevalence will continue to rise in the next years reaching 640 million adults by 2040 [2]. Over time, diabetes can trigger the development of several long-term associated conditions that contribute to a higher risk of morbidity and mortality. Such conditions can lead to a reduction of blood flow which, in combination with nerve damage, increases the chances of developing diabetic foot ulcers (DFU) [3]. DFU often heal very slowly and these open wounds provide a niche for colonization and infection [4]. Moreover, half of these ulcers will become infected non-healing foot ulcers, dramatically increasing the risk of gangrene and lower limb amputation [5]. Studies have shown that DFU usually presents polymicrobial infections; nevertheless, the most frequently identified organisms are *Staphylococcus aureus*, *Staphylococcus epidermidis*, coagulase-negative *Staphylococcus* spp., *Enterococcus* spp., *Escherichia coli*, *Pseudomonas aeruginosa*, *Proteus mirabilis*, and *Klebsiella pneumoniae* [6]. *S. aureus*, specially, methicillin-resistant *S. aureus* (MRSA), are opportunistic pathogens that can cause several types of infections from mild skin and soft-tissue infections to life-threatening endocarditis, such as, bacteremia, endocarditis, pneumonia, and bone and joint infections [7]. MRSA infections continue to be a major concern globally since these strains are usually resistant to several classes of antimicrobial drugs and often carry multiple virulence factors [8]. MRSA is often found in DFU in which the prevalence can range from 15 to 40% [9]. Although *S. aureus* is considered as a commensal organism and its colonization has benefits to the host such as inhibiting the growth of pathogenic organisms and regulating the host’s adaptive immune response, it can also promote chronic infections under certain conditions [10]. Therefore, *S. aureus* found in DFU may derive from the host commensal microflora or from an external source. Additionally, DFU provide a favorable environment for biofilm formation due to their suitable surface for cell attachment and the presence of adequate nutrients [11]. MRSA has a high capacity to form biofilms in DFU, shielding bacteria from the action of neutrophils, delaying wound healing and increasing their resistance to antimicrobial agents [12]. Thus, achieving healing of MRSA infected DFU has become a challenge. Methicillin-susceptible *S. aureus* (MSSA) are also frequently isolated from DFU and, in some studies, more frequently than MRSA [13,14]. Therefore, it is necessary to develop new potential antimicrobial agents to treat DFU infected by both MRSA and MSSA. Type 1 diabetes consists of the autoimmune destruction of pancreatic β cells. This type of diabetes can be triggered by several mechanisms such as the destruction of β cells by chemical agents, models that develop diabetes spontaneously, models that are genetically induced or by viruses [15]. Regarding the use of chemical agents for the induction of diabetes, this is a very effective method, which can be used in both small and large animals. The two most used agents are streptozotocin (STZ) and alloxane, which are selectively incorporated into β cells through GLUT 2 receptors and induce diabetes through two mechanisms: they inhibit glucokinase (the enzyme responsible for glucose phosphorylation) and they induce the formation of reactive oxygen species that cause the death of β cells [16]. The chemical induction method has the disadvantage that the chemical agents used are toxic to other organs of the animal, namely the liver, lung, kidney, intestine and brain [15]. Male rats are a common model to study diabetes due to the fact that they are more susceptible to STZ than female rats and since males do not have reproductive cycles or hormonal fluctuations that could interfere with the results [17]. Over the last few years, ozone has been introduced as an alternative to conventional antibiotics due to its recognized strong and effective antibacterial activity [18]. Other studies have reported the high antibacterial properties of ozone against MRSA strains [19]. Ozone acts on the cytoplasmic membrane, and due to its oxidant secondary effects, it induces modification of intracellular contents, such as oxidation of proteins and loss of organelle functions. Ozone improves cellular function and promotes wound healing since it enhances the formation of granulation tissue, accelerating the wound closure, mainly due to the increased oxygen tension within the wound [20]. Besides the antimicrobial and wound healing actions of ozone, it has also immunostimulating, analgesic, antihypoxic, detoxicating, bioenergetic and biosynthetic effects [21]. Nevertheless, studies have shown that ozone can also damage human cells and tissues [22,23]. Thus, we aimed to investigate the capacity of topical ozonated oil on the treatment of an MRSA abscess.

## 2. Materials and Methods

### 2.1. Ethics

Animal experiments were performed in strict accordance with the recommendations of the EU directive (2010/63/EU) for care and use of laboratory animals and National Legislation (Decreto-Lei 113/2013) for animal experimentation and welfare. Additionally, the experimental procedures were conducted under project license approval by the Organ Responsible for Animal Welfare (ORBEA) at University of Trás-os-Montes and Alto Douro and the Portuguese competent authority, Direção Geral de Alimentação e Veterinária (DGAV, Lisboa, Portugal) with reference number 0421/000/000/2019.

### 2.2. Ozonated Oils

Ozonated oil was prepared as previously described [19]. Briefly, 100 mL of a 50:50 mixture of extra virgin olive oil and refined sunflower oil was subjected to a gas stream of O2/O3 at a concentration of 75 µg/mL of ozone, in a continuous flow of 4 L/min and under normal pressure conditions. Ozone was generated using a Herrmann generator (HYpermedozon). Ozonated oil was exposed to the gas stream for 160 min producing an oil with a final concentration of ozone of 17.3 mg/g of oil. All oils were obtained from a single preparation. Oil mixture without ozonation was used in the control group.

### 2.3. Determination of the Peroxide Value

Peroxide value was determined according to the Portuguese Standard NP-904:1987 and as previously described [19]. Briefly, 10 mL chloroform, 1 mL potassium iodide saturated solution and 15 mL of glacial acetic acid were added to 0.5 g of oil. Then, the solution was mixed, and the sample was left 5 min in the dark. The liberated iodine was titrated with sodium thiosulfate 0.01 N. Peroxide value was expressed as milli-equivalents (meq) peroxide per 1 kg oil.

### 2.4. Bacterial Strain and Inoculum Preparation

One MRSA strain (EMRSA-15 clone) isolated from infected DFU and already characterized regarding its antibiotic resistance, virulence and molecular typing was used in all experiments [9]. The strain susceptibility to ozonated oil and the antibiofilm effect have also been previously investigated [19].

The strain was cultured in Brain Heart Infusion (BHI) agar and incubated at 37 °C for 24 h. After the incubation, a few colonies were suspended in BHI broth and incubated at 37 °C for 4–6 h to reach the log-phase of growth. The bacterial suspension was diluted to achieve 1 × 10^11^ colony-forming units per milliliter (CFU/mL). Density was measured using Ultrospec 10 Cell Density Meter instrument (Biochrom Ltd., Cambridge, England). Different bacterial concentrations were administrated in 3 animals prior to this study and it was verified that the animals inoculated with concentrations below 1 × 10^11^ did not develop infection.

### 2.5. Induction of Diabetes

To induce diabetes, 16 animals were injected with freshly prepared streptozotocin (STZ) (50 mg/kg). Three and eight days after the STZ administration, a drop of blood from the tail vein of each rat was tested using a commercial glucometer (GlucocardTM SM, Menarini diagnostics) to determine hyperglycemia development. All rats developed diabetes 8 days after induction.

### 2.6. Animals and Surgical Procedure

A total of sixteen Wistar adult female rats (Charles River Laboratories, Les Oncins, France), with an average weight of 300 ± 65 g, were used. All animals were kept at constant temperature and humidity (22 ± 1 °C and 55%, respectively) in controlled rooms with a 12/12 h light/dark cycle. The rats were housed in cages (1 animal) with corn kernels and received food pellets and water ad libitum. Animals were randomly assigned and blindly equally divided into two groups. Group I (control) received an application of oil in the wound twice a day for seven days. Group II (treatment) received an application of oil with ozone in the wound twice a day for seven days.

For surgical procedures, rats were anaesthetized in an anesthetic chamber with isoflurane at 5% for induction and maintained under isoflurane at 2% with mask. Pain control was achieved with buprenorphine by intraperitoneal injection (0.1 mg/kg). The back of the animal was shaved, disinfected with chlorhexidine and dried. Skin inoculation was performed with an intradermal injection of 10 µL of the MRSA suspension in the midline approximately 1 cm behind the neck, producing an abscess lesion. After 3 days the skin epidermis was cut and removed with a 6 mm biopsy punch in order to open the wound and remove the pus with a sterilized swab soaked in sterilized saline solution to clean the wound, followed by the application of the treatment option. Cleaning and treatment procedures were performed for 7 days, twice a day, under isoflurane anesthesia. The oil was applied with a dropper (3 drops per application and about 40 µL per drop). During the treatment period, analgesia was obtained using meloxican by subcutaneous injection (1 mg/kg) once a day.

### 2.7. Sacrifice

At the end of the seven-day treatment period, animals were sacrificed applying a lethal dose of pentobarbital (80 mg/kg) by intracardiac injection, under general anesthesia. The skin of each animal was collected from the lesion with a 10 mm biopsy punch and then the skin disks were inserted into a sterile Eppendorf with 2 mL of sterile saline solution for microbiological analysis.

### 2.8. Microbiological Evaluation

Signs of infection and hound healing were evaluated at the end of the experiment macroscopically by the presence of local reaction and pus formation and microscopically by histological examination.

Tubes containing the skin samples were subjected to vortex mixing and sonicated at a frequency of 35 kHz in a water bath to detach bacteria from the skin surface. After sonication, 5 serial dilutions of the saline solution containing the skin samples were performed. One hundred microliters of the bacterial suspension from each tube was plated on Plate Count agar plates and incubated at 37 °C for 24 h. After incubation, the number of bacterial CFUs was determined. The presence of MRSA was confirmed by Gram-staining, colony morphology and biochemical tests, including, catalase, DNase and coagulase tests [24].

### 2.9. Histopathological Evaluation

To evaluate wound healing, histological examinations were carried out to assess cellular responses and vascularization. Wound samples were fixed with a 10% formaldehyde buffer solution for 3 days. Then, skin samples were dehydrated by a series of increasing ethanol concentrations, processed in xylene and embedded in paraffin. The tissue was sectioned using a histological microtome. Section 3 and Section 4 mm thick were stained using haematoxylin and eosin (H&E). A semi-quantitative method was performed blindly to examine epidermal regeneration, granulation tissue thickness, fibroblast proliferation, angiogenesis and the presence of inflammatory cells as previously described [25]. The stained sections were scored using a scale from 0 to 4 for angiogenesis, fibroblast proliferation and the presence of inflammatory cells; and from 1 to 3 for granulation tissue thickness and epidermal regeneration [25].

### 2.10. Statistical Analysis

The quantitative culture results are presented as mean ± standard deviation (SD). Statistical comparisons between the groups were performed by the independent samples *t*-test. Differences were considered significant when metabolite *p* < 0.05. All analyses were performed using IBM SPSS statistic for Mac, version 26.0 (IBM Corp., Armonk, New York, NY, USA).

## 3. Results

**Figure 1:**
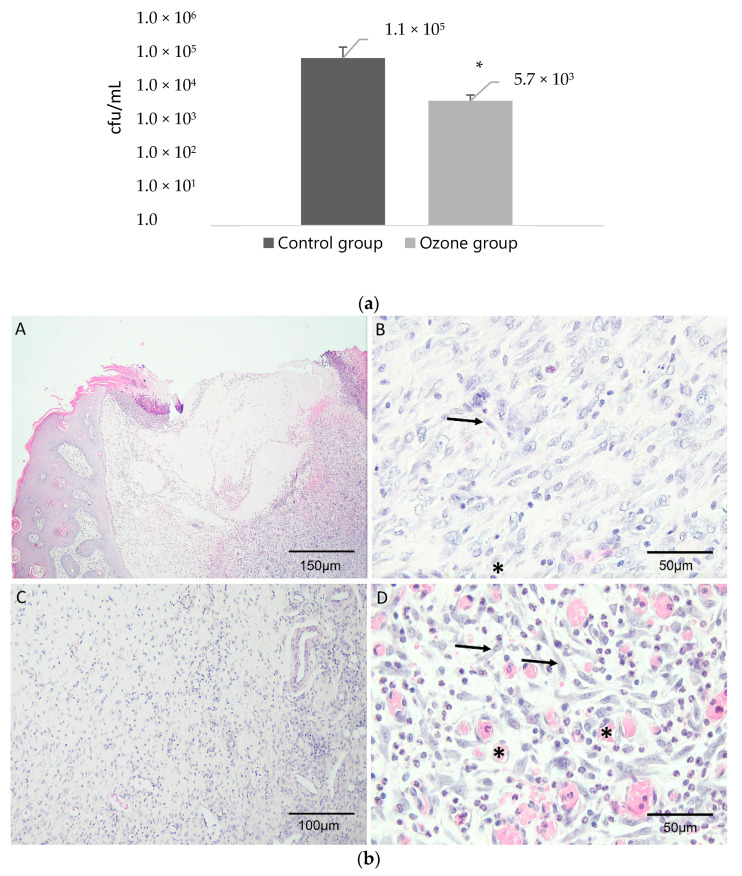

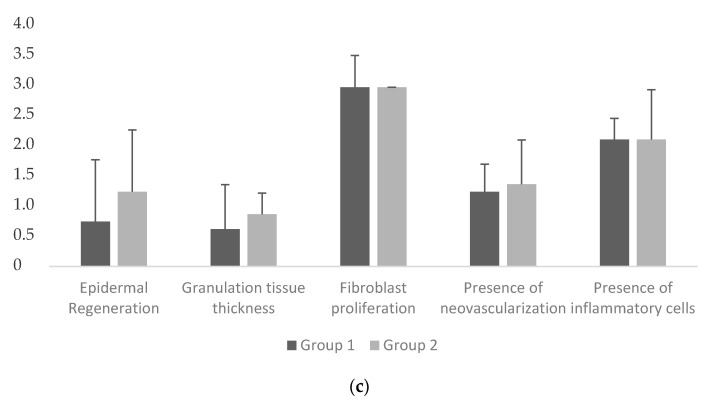
(**a**) Mean and standard deviation of cfu/mL in rats from control groups and ozone treatment group. * statistically difference from group 1 (*p* < 0.05). (**b**) Images of the healed wound site from a rat from Group 1 (control group) (**A**,**B**) and a rat from Group 2 (ozone group) (**C**,**D**). Rats treated with ozonated oil show more granulation tissue (**C**) and neovascularization (**D**) compared with no treated rats. Representation: asterisk (*): angiogenesis, arrow (→): fibroblast cell. (**c**) Histological score of epidermal regeneration and granulation tissue thickness scored from 0 to 3; and fibroblast proliferation, angiogenesis and the presence of inflammatory cells scored from 0 to 4.

Vegetable oils were ozonated and their content in peroxides was evaluated. Diabetes was induced in 16 Wistar rats by an intraperitoneal injection of STZ. Three days after STZ administration the glucose was measured, and the animals had an average of 130.5 mg/dL. The level of glucose was measured again 8 days after STZ administration and 11 out of 16 rats had a concentration above 600 mg/dL and the remaining five animals presented an average of 522 mg/dL. The 16 rats were divided into two groups and inoculated by an intradermic injection of MRSA suspension, producing an abscess lesion. Group 1 received an application of oil mixture without ozone treatment and Group 2 received an application of ozonated oil. After the treatment period, skin was collected, colony-forming units (CFU) of bacteria were quantified and the histological analysis of the skin was carried out. The peroxide value of the two oils used in this study was determined. The control group received treatment with a mixture of extra virgin olive oil and refined sunflower oil which had a very low peroxide value of 3.9 meq O_2_/kg when compared to the oil used in the treatment group/113.5 ± 3.7 meq O_2_/kg). Macroscopically, at the end of the experiment, animals from group 1 showed some external signs of infection whereas animals from group 2 did not show any external signal of infection. Regarding the persistence of infection, in group 1 (control group) the bacterial load was 1.1 × 10^5^ cfu/mL revealing a well-established infection which is indicative that the vegetable oil itself had no effect on the infection eradication. Group 2 had a significantly lower bacterial load than the control group of 5.7 × 10^3^ cfu/mL with a level of significance of 0.028 (Figure 1). Macroscopically, wounds of animals treated with ozonated oil were completely healed in seven out of eight rats while three animals from group 1 still presented open wounds which shows the already proven wound healing efficacy of ozonated oils. Histological evaluations were conducted to study the response of cellular wound repair mechanisms from the application of ozonated oils and the results are presented in Figure 1. Both groups presented an incomplete reepithelialisation and poorly formed granulation tissue. The histological analysis showed improvement, although small, in the healing process for Group 2. In group 1 (control), three of the eight animals did not show any epidermal regeneration, three animals showed mild regeneration and one animal showed moderate regeneration. In group 2 (ozone group), all the animals showed epidermal regeneration, moderate in two animals and mild in six animals. Regarding the granulation tissue thickness, animals from group two had also higher scores than animals from group 1. Half of the control animals had no granulation while in the ozone group only one animal showed no granulation tissue. Angiogenesis is generally occasionally present, but in two animals of the ozone group was light scattering or abundant. The score of fibroblast formation and the presence of inflammatory cells were similar in the two groups.

## 4. Discussion

Ozonated vegetable oils are widely recognized for their wound healing properties and antimicrobial activity and have been used in cosmetics, food applications, disinfection and pharmaceutical industries [26,27,28]. The activity of ozonated oils is due to the products of the reaction of ozone with the carbon–carbon double bonds of the unsaturated fatty acids in vegetable oils. Among these products, the most significant are trioxolanes and peroxides [29]. The peroxide value represents the amount of peroxides in an oil sample and so, it is essential to determine the peroxide value in order to establish the therapeutic dose. The peroxide value of a mixture of extra virgin olive oil and refined sunflower oil was 3.9 meq O_2_/kg whereas the ozonated oil was 113.5 ± 3.7 meq O_2_/kg. Studies have shown that ozonated oils with higher peroxide values have a better effect on the acceleration of wound healing and higher antibacterial activity [30,31]. Ozone gas and both ozonated oil and water have proven activity against *S. aureus* and MRSA strains. Several studies have shown the bactericidal potential of ozone in vitro [18,31,32]. Furthermore, other studies have reported the efficacy of ozone on infection eradication in vivo [32,33]. However, under diabetic conditions, wound healing and eradication of infection are harder to accomplish. Infections in diabetic patients are more frequent and difficult to treat than in non-diabetic individuals mainly due to neuropathy, and arteriopathy and immunodeficiency [34]. Therefore, in this study, we evaluate the ozonated oil capacity to eradicate MRSA infection in an animal model of diabetic rat. Two groups of animals were used: in group 1 (control group) the diabetic animals were treated with topical application of the oil mixture without ozonation and in group 2 the animals were treated with the ozonated oil at a concentration of 17 mg/g. After 7 days of treatment, all animals were euthanized, and the microbiological evaluation of the infected area was performed. In order to verify if there was contamination of the infection site, the recovered bacteria were subjected to several biochemical tests which all revealed that it was the bacterial strain used for induction of infection. Regarding the persistence of infection, animals from Group 1 had a higher bacterial load than animals from Group 2. The use of antimicrobials in the earlier stages of the DFU infection can be replaced by other treatments. Furthermore, the treatment of a DFU infection must be addressed according to the bacterial species in the wound and when the DFU has a high bioburden, topical antimicrobials can lead to a decrease of the bacterial load [35,36]. Although the action of ozonated oils in tissues is not fully characterized, some studies have reported that ozone triggers a cascade of reactions that results in a more efficient wound healing due to oxygen diffusion, upregulation of antioxidants enzymes and oxygen diffusion [37,38,39,40]. In our study, animals from Group 2 showed a mild to moderate epidermal regeneration and a greater granulation tissue thickness than animals from Group 1. Studies have shown that oxygen tension in wounds and their treatment with ozone may increase the formation of granulation tissue; therefore, the accelerated trend of wound closure shown in group 2 may be due to the exposure to ozonated oil together with decreased bacterial load found in this group [41,42]. There are studies reporting the effect of ozone in foot ulcers in vivo and there are also studies reporting the antibacterial activity of ozone in vivo. However, none of those studies investigated the infection eradication capacity of ozonated oils in DFU. Furthermore, most studies focus only on the wound healing effect of ozone. Therefore, as far as we know, this is the first study investigating the efficacy of ozone in the eradication of DFU infected by MRSA. A study conducted by Song et al. (2018) reported the high efficacy of ozone on the healing MRSA induced skin ulceration in non-diabetic patients [32]. In the study by Zhang et al. (2014), ozone treatment significantly promoted the early effective rate of wound healing in DFU of non-diabetic patients [43]. It has been shown that ozonated oils are a reservoir of ozone that, after topical application, is slowly released to the skin [44]. This feature can be useful for the topical use of ozone to treat infected DFU. Although *S. aureus* is the pathogen most frequently isolated from DFU, these lesions are often polymicrobial [45]. Therefore, it is important to consider both microbiological and clinical features to select the appropriate antibiotic therapy [13]. Nevertheless, studies have shown that ozone application leads to a reduction of biofilms formed by several different microorganisms. Elshinawy et al. (2018) tested the efficacy of ozonated olive oil on the removal of biofilm biomass of mixed-species biofilms and demonstrated a 79% reduction of biomass [46]. Tonnon et al. (2020) showed that the anti-biofilm effect of ozonized saline solution was similar to the effect of chlorhexidine in multi-species biofilms [47]. However, studies have shown that resistance to ozone may occur in bacteria due to N-acetyl glucosamine that is present in the peptidoglycan cell wall [48,49].

## 5. Conclusions

Topical application of ozonated vegetable oil in MRSA-infected skin ulcers, mimicking the DFU, in rats showed a significant reduction of the bacterial load and a macroscopically wound healing when compared to non-treated animals. However, the infection was not totally eradicated which may be due to the short treatment period further studies will be carried out evaluating the effect of different periods on treatment. Given the current emergence of antibiotic-resistant bacteria, especially in DFU, ozone therapy may represent a potential alternative or a coadjutant for the treatment of infected wounds and DFU avoiding the overuse of conventional antimicrobials. Further studies will be carried out regarding the efficacy of ozonated oils on the eradication of bacterial biofilms in vivo.
